# Impact of *Meyerozyma guilliermondii* isolated from chickens against *Eimeria* sp. protozoan, an *in vitro* analysis

**DOI:** 10.1186/s12917-015-0589-0

**Published:** 2015-11-09

**Authors:** Edgar Dantán-González, Rosa Estela Quiroz-Castañeda, Mayra Cobaxin-Cárdenas, Jorge Valle-Hernández, Yitzel Gama-Martínez, José Raunel Tinoco-Valencia, Leobardo Serrano-Carreón, Laura Ortiz-Hernández

**Affiliations:** Laboratorio de Investigaciones Ambientales-Centro de Investigación en Biotecnología, Universidad Autónoma del Estado de Morelos, Cuernavaca, Morelos Mexico; Unidad de Anaplasmosis del CENID-Parasitología Veterinaria, Instituto Nacional de Investigaciones Forestales, Agrícolas y Pecuarias, Juitepec, Morelos Mexico; Facltad de Ciencias Biológicas, Universidad Autónoma del Estado de Morelos, Cuernavaca, Mexico; Unidad de Escalamiento y Planta Piloto-Instituto de Biotecnología, Universidad Nacional Autónoma de México, Cuernavaca, Morelos Mexico

**Keywords:** Avian coccidiosis, Oocyst, *Meyerozyma guilliermondii*, Anticoccidial

## Abstract

**Background:**

Avian coccidiosis is a disease caused worldwide by several species of parasite *Eimeria* that causes significant economic losses. This disease affects chickens development and production, that most of times is controlled with anticoccidial drugs. Although efforts have been made to address this disease, they have been made to control *Eimeria* sporozoites, although enteric stages are often vulnerable, however; the parasite oocyst remains a problem that must be controlled, as it has a resistant structure that facilitates dispersion. Despite some commercial products based on chemical compounds have been developed as disinfectants that destroy oocysts, the solution of the problem remains to be solved.

**Results:**

In this work, we assessed *in vitro* anticoccidial activity of a compound(s) secreted by yeast isolated in oocysts suspension from infected chickens. The yeast was molecularly identified as *Meyerozyma guilliermondii,* and its anticoccidial activity against *Eimeria tenella* oocysts was assessed. Here, we report the damage to oocysts walls caused by *M. guilliermondii* culture, supernatant, supernatant extract and intracellular proteins. In all cases, a significant decreased of oocysts was observed.

**Conclusions:**

The yeast *Meyerozyma guilliermondii* secretes a compound with anticoccidial activity and also has a compound of protein nature that damages the resistant structure of oocyst, showing the potential of this yeast and its products as a feasible method of coccidiosis control.

**Electronic supplementary material:**

The online version of this article (doi:10.1186/s12917-015-0589-0) contains supplementary material, which is available to authorized users.

## Background

Coccidiosis is a widespread disease reported in numerous vertebrate that is caused by *Eimeria* species and shows strict host specificity [1]. In the poultry industry, which raises approximately 40 billion chickens annually, coccidiosis represents a serious disease that results in annual global economic losses of approximately $2.4 billion, including production losses and prevention and treatments costs [2–4].

Chicken coccidiosis is caused by *Eimeria* species that belongs to Apicomplexa, a phylum that includes veterinary and medical significance protozoa such as *Cryptosporidium*, *Neospora*, *Plasmodium*, and *Toxoplasma* [2]. *Eimeria* invade and destroy the intestinal epithelium of chickens, as a consequence infected birds display reduced feed intake, bloody diarrhea and hampered weight gain [5, 6].

Coccidiosis in chickens is a complex disease caused by one or more of several *Eimeria* species [7].

It has been reported that about 1800 *Eimeria* spp can affect the intestinal mucosa of mammals and birds, but only seven species are considered the causative agent of avian coccidiosis: *E. tenella*, *E. necatrix, E. acervulina, E, maxima, E. brunetti, E. mitis* and *E. praecox* [8].

*Eimeria* spp. has a complex life cycle, during which a resistant bilayered structure called oocyst is formed. The oocyst is non-infective when is unsporulated inside the chicken turning to infective when is sporulated, which occurs in the environment [2, 9].

The oocyst is considered a remarkably hard and persistent structure due to its composition of proteins, carbohydrate and lipids, which varies among species. It is also relatively resistant to mechanical and chemical damage, and to proteolytic degradation [10–12].

Coccidiosis disease control efforts have been focused on the development of several anticoccidials (ionophores and synthetic chemicals) that have been mainly directed to the parasites during the sexual and asexual stages that occur within the host rather than targeting the most infectious stage, the oocyst [13].

Due to anticoccidial drug resistance observed in birds around the world, a search for natural products with anticoccidial action has emerged, including fungi, plants and essential oils (oregano, laurel leaf, lavender, artemisia, clove, and tea tree) [14–18]. Recently, [19] described a list with natural products commercially available.

Despite the anticoccidial activity observed for natural products, the elevated cost of farming and production required to obtain sufficiently large quantities make their use impractical as a strategy to control coccidiosis in large population of birds.

With the aim of finding effective anticoccidial products that can be an alternative to anticoccidial reported, in this work, we isolated a yeast from an oocyst suspension from infected chickens and identified by molecular methods as *Meyerozyma guilliermondii* 01. We observed a surprising anticoccidial activity in the yeast culture, yeast culture medium supernatant and ethyl acetate extract of the supernatant, all have the ability to break oocysts, the strongest structures made by different species of *Eimeria.* Interestingly, the damage caused by *M. guilliermondii* 01 involves destruction of the oocyst wall and weakening of its structure, and thus reducing the probability of infection. The newfound anticoccidial activity of *M. guilliermondii* 01 might have implications for the development of novel anticoccidial control drugs.

## Methods

### Isolation of *Meyerozyma guilliermondii*

The yeast *M. guilliermondii* was isolated from oocyst suspensions recovered from ATD-1Coccivac B (MSD Animal Health), a commercial vaccine of live oocysts (*E. acervulina, E.mivati, E.maxima* and *E. tenella*). This vaccine is produced by Merck, a company that is intended to use ethically resources to fulfill customers expectative, they are adhere to: the International Federation for Animal Health Statement of Principles; the US-bases Animal Health Institute Advertising Guidelines; The UK-based National Office of Animal Health Code of Practice for the Promotion of Animal Medicine.

A loopful of a suspension was used to inoculate Petri dishes (90 × 15 mm, Ruisanchez, Mexico) of YPDA medium (Yeast extract, Peptone, Dextrose, Agar) [20 g/L yeast extract (BD, USA), 5 g/L peptone (BD Difco, USA), 40 g/L dextrose (Fermont, Mexico), 10 g/L agar (BD, USA)]; the plates were incubated (LabTech 3016A, USA) at 28 °C for 16 h. After the incubation, yeast colonies were observed, picked and grown in liquid YPD (Yeast extract, Peptone, Dextrose) [20 g/L yeast extract (BD, USA), 5 g/L peptone (BD Difco, USA), 40 g/L dextrose (Fermont, Mexico)] under the same conditions for further identification. Solid YPD was used only to grow and preserve yeast for a short period of time at 4 °C on a Petri dish. A loopful from this plate was used to cultivate *M. guilliermondii* in liquid YPD to perform the bioassays.

### Obtaining of oocysts used in bioassays

*Eimeria tenella* oocyst suspensions were obtained from infected chickens and maintained in potassium dichromate at 4 °C. *E. tenella* oocysts were kindly provided by SENASICA (Servicio Nacional de Sanidad Inocuidad y Calidad Agroalimentaria,). All protocols used in this study were approved by the animal ethics committee of National Commission on Bioethics (http://www.conbioetica-mexico.salud.gob.mx/) consistent with the recommendations of the Mexican Official Standard NOM-062-ZOO-1999, Technical Specifications for the production, care and use of laboratory animals.

*Eimeria* spp. oocysts were obtained from commercial vaccine Autocox (Eimeria, Mexico) that contains oocysts from *E. acervulina, E. maxima, E. tenella* and *E. praecox.* Eimeria S.A de C.V., is certified by Secretaría de Agricultura, Ganadería, Desarrollo Rural, Pesca y Alimentación (SAGARPA, México) since it fulfills the requirement of good practices in manufacture of the vaccines contained in NOM-022-ZOO-1995 y NOM-026-ZOO-1994.

### Amplification by PCR of 5.8S-ITS rDNA region of *M. guilliermondii*

Yeast cells were grown on YPDA medium at 28 °C for 48 h. Cells were collected with the tip of a toothpick and directly used for polymerase chain reaction (PCR).

To amplify the 5.8S-ITS rDNA region and the D1/D2 domain, a PCR reaction was performed as reported by [20]. Thermal cycling was conducted with an initial step at 95 °C for 5 min, 40 cycles of 94 °C for 40 s, 55 °C for 40 s and 72 °C for 30 s and a final extension of 10 min at 72 °C.

Amplification of the 5.8S-ITS rDNA region was achieved with the primers reported by [21]: its1 (5′-TCCGTAGGTGAACCTGCGG-3′) and its4 (5′-TCCTCCGCTTATTGATATGC-3′). Amplification of the D1/D2 domain of the 26S rRNA gene was achieved with the primers NL1 (5′GCATATCAATAAGCGGAGGAAAAG-3′) and NL4 (5′-GGTCCGTG TTTCAAGACGG-3′) [22].

After PCR, the 620-bp amplification products were excised from a 1 % agarose gel and purified using a High Pure PCR Product Purification Kit (Roche, USA) according to the manufacturer’s instructions. The purified fragments were then sequenced (Unidad de Síntesis y Secuenciación de AND, Instituto de Biotecnología-UNAM), and a BLASTn analysis was performed (http://blast.ncbi.nlm.nih.gov/Blast.cgi). In order to detect nucleotide mutations and to confirm the integrity of the sequence, we have sequenced five independent clones.

### *In silico* restriction fragment length polymorphism (RFLP) of 5.8S-ITS rDNA sequences

Once the purified fragment 0f 620 bp were sequenced, an in silico enzyme digestion with *Cfo*I, *Hae*III and *Hin*fI was performed with EnzymeX software and Restriction Mapper V3 (http://www.restrictionmapper.org/).

### Amplification by PCR of 18S rRNA

To amplify a 1565-bp product corresponding to sequence of 18S rRNA of *M . guilliermondii* (GenBank accession number KJ126853), we designed the primers Mgfwd: 5′AAACTGCGAATGGCTCATTAAATCAGTTATCG-3′ and Mgrev: 5′-GCGACGGGCGGTGTGTACAAAGG -3′. Thermal cycling was conducted with an initial step at 95 °C for 5 min, 35 cycles of 94 °C for 40 s, 61 °C for 1 min and 72 °C for 1.5 min and a final extension of 10 min at 72 °C.

### Phylogeny reconstruction

A phylogenetic reconstruction was performed with the 1565 bp sequence of the 18S rDNA from *M. guilliermondii* 01 (GenBank accession number KJ126853) and 20 sequences retrieved in a BLASTn analysis.

The phylogeny was constructed with the software tool MEGA (Molecular Evolutionary Genetics Analysis) 6.0 [23], with the method Neighbor-joining and the predicted model of Maximun Compositum Likelihood. The robustness of the trees was statistically evaluated by bootstrap analysis with 1000 iterations.

### Growth of *M. guilliermondii 01*

A loopful of yeast cells was grown in a pre-inoculum of 20 ml YPD for 14 h at 30 °C and 200 rpm (LabTech 3016A, USA). Next, 100 ml of YPD was inoculated with a starting optical density at 600 nm (OD_600_) (Thermo Biomate 3 IV-Vis Spectrometer, USA) of 0.1 and grown until the OD_600_ reached 1.0 or 1.8 (approximately 8 h) under the same temperature and shaking conditions. An OD_600_ of 1.0 corresponds to 2.72 × 10^6^ CFU/ml, and an OD_600_ of 1.4 corresponds to 6.72 × 10^6^ CFU/ml.

The effect of different CFU/ml values of *M. guilliermondii* on oocyst integrity was assessed by bioassays.

### Recovery of culture and supernatant of *M. guilliermondii*

A culture of *M. guilliermondii* was obtained as described above, and 200 μl of the culture was used in each bioassay. For bioassays using the supernatant, supernatants were obtained by centrifugation at 4 °C for 15 min at 3,500 *g* (17303-15, Cole Parmer, USA), and 200 μl was used for anticoccidial activity bioassays. 200 μl of non-inoculated liquid YPD was cultivated in the same mentioned conditions and used in bioassays reactions. The supernatant of YPD medium was recovered by centrifugation and also used as negative control.

### Ethyl acetate extraction of supernatant

*M. guilliermondii* culture supernatant (100 ml) was extracted three times with ethyl acetate (High Purity, Mexico). Briefly, the culture was mixed with one volume of ethyl acetate and strongly shaken by hand in an extraction funnel to homogenize the mixture. The funnel was then placed in a universal mount until two phases were observed (approximately 3 to 5 min). The upper phase was recovered and further extracted twice.

The final extraction volume (approximately 300 ml) was evaporated in a rotary evaporator (R-205, Buchi, Switzerland) at 80 rpm and 41 °C. The extract was then recovered in amber vials, dried for 4 days in an extractor hood, weighed and dissolved in approximately 20 to 30 μl of dimethyl sulfoxide (DMSO, J.T. Baker, USA). Two milligrams of the supernatant extract was used in anticoccidial activity bioassays. Non-inoculated YPD was also extracted with ethyl acetate and concentrated by evaporation in the same conditions. 2 mg of the product of this extraction resuspended in DMSO were used as negative control in bioassay reactions. In order to assess the nature of the compound extracted with ethyl acetate we performed incubation with trypsin (aqui no se las condiciones de tiempo y cantidad de enzima en que lo hizo Mayra).

### Intracellular proteins extraction

Yeast cell were grown in the conditions mentioned above. After this, the culture was centrifuged for 15 min at 9,000 g and 4 °C and the supernatant was discarded. The pellet was recovered and resuspended in 20 mM phosphate buffer (pH 7.0). The cells were lysed by sonication for 10 min and 32 % of amplitude (Ultrasonic processor, Sonic & Materials) and then centrifuged for 15 min at 9,000 g and 4 °C.

The recovered supernatant, that contained the intracellular crude extract, was filtered through a PVDF membrane (Millex GV filter unit 0.22-μm, Millipore) and then quantified by the Bradford assay [24] using a standard curve of bovine serum albumin (BSA).

### Proteins precipitation with ammonium sulfate

The concentrated intracellular proteins were precipitated with 20, 40, 60 and 80 % of saturated solution of ammonium sulfate. This mix was stirred on ice for 2 h and then centrifuged at 15,000 g for 15 min at 4 °C. The supernatant was discarded and the pellet was dissolved in 20 mM phosphate buffer (pH 7.0) and dialyzed overnight (12 kD dialysis tubing membrane, Sigma-Aldrich). Finally, the dialyzed fractionated proteins were concentrated by centrifugation (Amicon Ultra Concentrator with 10 kD molecular mass cut-off, Millipore) and quantified by the Bradford assay [24] using a standard curve of bovine serum albumin (BSA). Each fraction was used in anticoccidial activity bioassays.

### Proteins denaturalizing treatment

Five hundred micrograms of proteins precipitated with a 40 % saturated solution of ammonium acetate were treated with a 1/10 of trypsin (1 mg/ml, Sigma-Aldrich) diluted in 12.5 mM Tris-HCl (pH 9.0) for 12 h at 37 °C. After this, proteins were incubated for 10 min at room temperature and then 10 min on ice. To stop the reaction, 100 mM of PMSF (phenylmethanesulfonylfluoride) protease inhibitor (Sigma-Aldrich) was added in a 2 mM final concentration; the reaction was mixed by inversion and kept on ice until use.

For samples treated with trichloroacetic acid (TCA), 1,500 μg of 40 % fractionated proteins were precipitated with 1 volume of cold TCA 20 % and mixed by inversion before incubate 20 min on ice. The mix was agitated by inversion several times and then centrifuged at 15,000 g for 15 min. The pellet was washed three times with 500 μl of cold acetone with intervals of centrifugation at 15,000 g for 10 min. Finally, once the acetone was evaporated; the pellet was resuspended in 10 mM Tris-HCl to neutralize the pH and 20 mM phosphate buffer (pH 7.0) was added to a final volume of 1 ml.

Separately, 1,500 μg of the 40 % fraction recovered after ammonium sulfate precipitation were heated for 15 min at 95 °C and then kept on ice for 15 min.

Both samples, treated with TCA and heat, were quantified and assayed for anticoccidial activity.

### *In vitro* anticoccidial activity bioassays

Bioassays were performed using *E. tenella* oocyst suspensions (SENASICA) and *Eimeria* spp. commercial oocyst suspensions (ATD-1Coccivac B). Both oocysts suspensions consisted of a heterogeneous mixture of unsporulated and sporulated oocyst, with a predominance of sporulated (infective).

Oocysts were washed and quantified in a Neubauer chamber (Improved Bright Line, Loptik Labor, Germany) before bioassays were performed. A 1 ml sample of oocysts was washed three times with 1 ml of sterile injectable water (Pisa, Mexico) until the medium was clarified; centrifugation at 850 *g* for 1 min (Thermo Electron IEC Micromax Microcentrifuge, USA) was performed for every wash.

Under sterile conditions, bioassay reactions were prepared in 1.5 ml Eppendorf tubes containing 100,000 *E. tenella* oocysts along with 200 μl of *M. guilliermondii* culture, 200 μl of supernatant, 2 mg of ethyl acetate supernatant extract, 300 μg of intracellular proteins, 300 μg of 40 % fraction precipitated with ammonium sulfate or 300 μg of 40 % fraction treated with trypsin.

For the bioassays performed with culture and supernatant the final volume was 1 ml adjusted with injectable water. Bioassay reactions with ethyl acetate supernatant extracts were prepared in 1.5-ml tubes with 100,000 oocysts and 2 mg of extract dissolved in DMSO (final concentration 2 %, 20 μl), and the reactions were brought to 1 ml with injectable water as is shown in Additional file 1. On the contrary, all bioassays performed with intracellular proteins and 40 % fractions (with or without trypsin) were adjusted to 1 ml with 20 mM phosphate buffer (pH 7.0). Bioassays reactions were incubated at 30 °C and 200 rpm (LabTech 3016A, USA) and then observed microscopically after 24 and 48 h of incubation. Oocysts from the reactions were counted in a Neubauer chamber at 0, 24 and 48 h. Oocyst integrity was assessed microscopically (Leica DM750), an oocyst wall without damage was considered as a viable and infective oocyst, whereas an oocyst wall with the slightest damage was considered as non-viable and therefore non-infective oocyst.

The amount, 2 mg of ethyl acetate extract allows observing clearly the damage of the extract on oocysts in a determined period of time. Higher amounts of compound causes that damage in oocysts is not accurately observable; lower amounts extends the time of observation and damage is barely noticeable in a determined period of time.

Depending on the case, control reactions were prepared with only 100,000 oocysts, 200 μl of YPD medium, or supernatant of YPD medium and injectable water (up to 1 ml). As regards to control reaction in bioassays containing ethyl acetate extract, it contained, 100,000 oocysts, ethyl acetate extract of YPD medium resuspended in DMSO and injectable water (up to 1 ml). Control reaction of intracellular crude extract and 40 % ammonium sulfate fractions contained 100,000 oocysts and phosphate buffer 20 mM pH 7.2 (up to 1 ml). Control reactions were incubated under the same temperature and shaking conditions.

All experiments were standardized with respect to controls, so the standard deviation is not plotted. Independent triplicates were performed for each bioassay.

### Statistical analysis

All statistical analyses were performed with Minitab® 15 Statistical Software. A one-way analysis of variance was used to analyze the differences in mortality among the different bioassays. To determine significant differences among the means, a Tukey test was performed. The significance threshold was set at *P* < 0.05.

## Results

### BLAST analysis

We performed a BLASTn search with the *M. guilliermondii* 5.8S-ITS rDNA and D1/D2 domain sequences to find similarities with reported sequences.

The 620-bp 5.8S-ITS rDNA query sequence showed 100 % sequence identity with *Pichia guilliermondii* (FJ515181.1), 99 % with *Meyerozyma guilliermondii* (JQ425356.1, HQ693808.1) and 99 % with *Pichia guilliermondii* (EF222226.1, 197816.1).

A similar result was obtained for a 632–bp D1-D2 domain sequence (GenBank accession KJ126855). BLASTn analysis showed 99 % sequence identity with *Pichia guilliermondii* (EJ515181.1, FJ969194.1, EU569871.1) and *Meyerozyma guilliermondii* (JQ425356.1, HQ693807.1). The percentages of query coverage in these analyses ranged from 98 to 99 %.

### *In silico* restriction analysis of 5.8S-ITS rDNA

An *in silico* enzymatic digestion of the 620-bp sequence (GenBank accession number KJ126854) corresponding to the 5.8 ITS rDNA region was performed with *Cfo*I, *Hae*III and *Hin*fI, and all the predicted fragments coincided with those reported by [20].

EnzymeX and RestrictionMapper digestion predicts *Cfo*I fragments of 314 and 255 bp, *Hae*III fragments of 392, 108 and 87 bp and *Hin*fI fragments of 335 and 277 bp.

### Molecular identification of *Meyerozyma guilliermondii 01*

A 1565-bp sequence from the 18S rDNA region of *M. guilliermondii* (GenBank accession number KJ126853) was used to construct a phylogeny with 20 sequences retrieved by BLASTn. Sample results from this analysis include 100 % identity with *M. guilliermondii* (KC178873.1) and *P. guilliermondii* (DQ821711.1), 99 % identity with *P. caribbica* (EF194890.1).

**Fig. 1:**
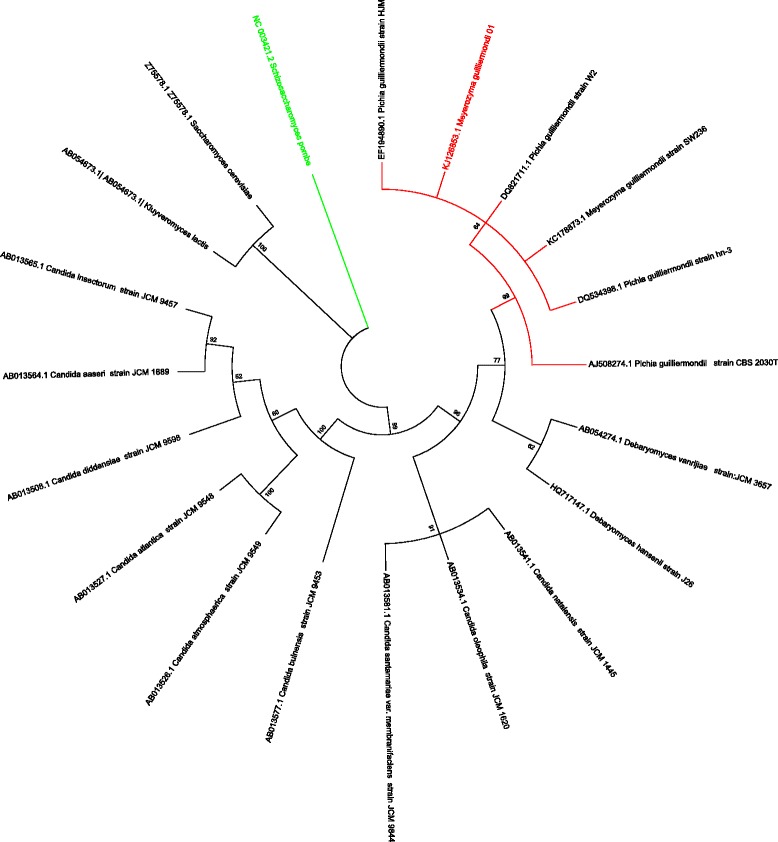
Phylogenetic tree of *M. guilliermondii* 01 based on the 18S molecular marker. *M. guilliermondii* 01 is grouped with *P. guilliermondii* and *M. guilliermondii*, which strengthens the identity of the yeast. Phylogeny reconstruction was performed with MEGA 6.0 and 1000 replications. Bootstrap values are shown in branches nodes

The phylogenetic tree shows that the yeast isolated from oocysts is grouped with *P. guilliermondii* and *M. guilliermondii.* A representative tree is shown in Fig. 1. We followed the new genera proposed by Kurtzman and Suzuki [25], in which the authors indicate that the species *P. guilliermondii* and *P. caribbica* now belong to the genus *Meyerozyma.*

### Dose-effect analysis of *M. guilliermondii* 01 on *Eimeria* spp. oocysts

**Fig. 2:**
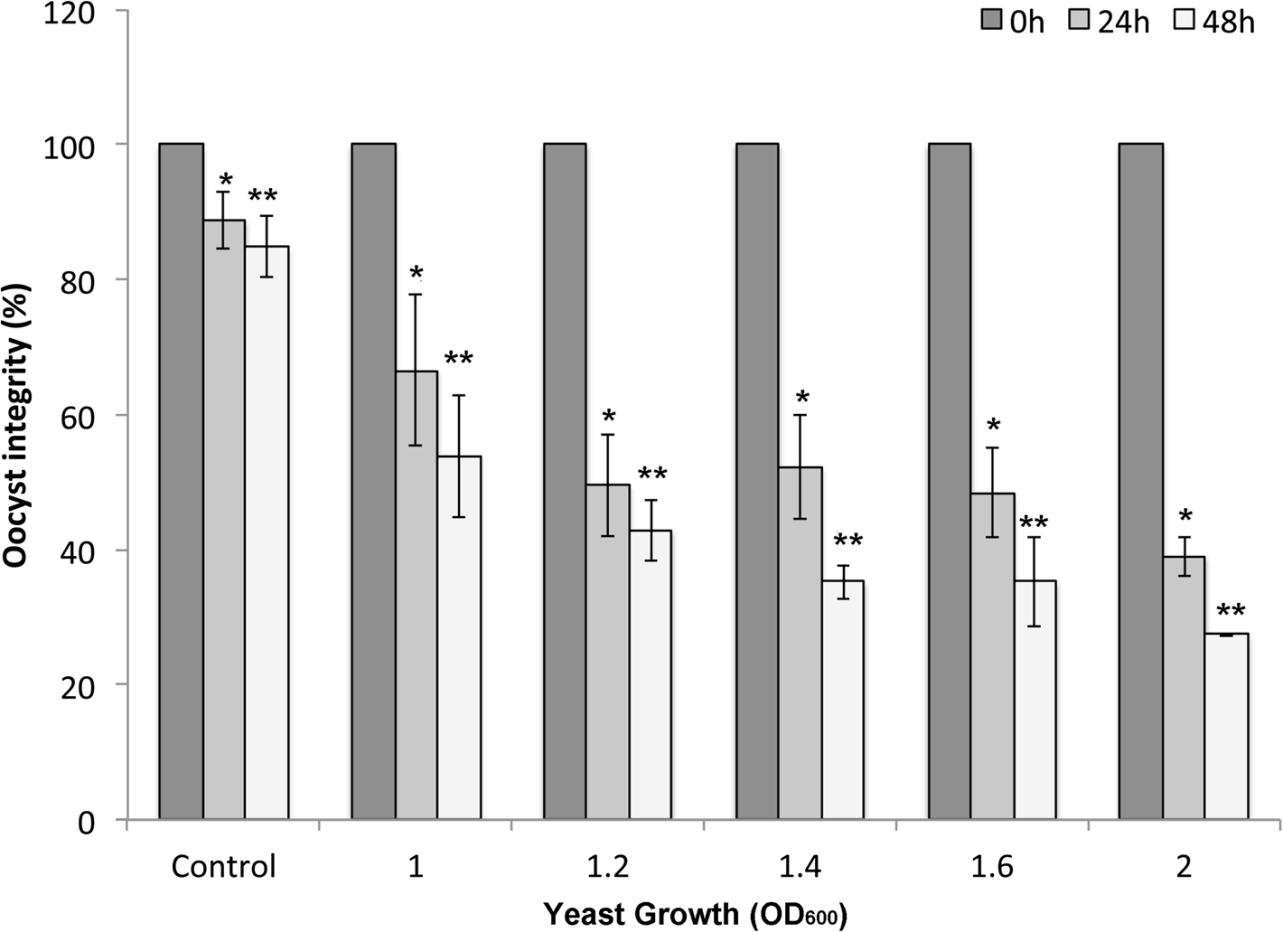
Anticoccidial activity *M. guilliermondii* 01 on *E. tenella* oocysts at increasing optical densities (OD_600_). After 48 h, with a higher number of cells at OD_600_, a more lethal anticoccidial activity was observed. Significant differences among control and 24 h are shown with an asterisk and significant differences among control and 48 h are shown with double asterisk (significance threshold, *P* < 0.05)

Bioassays performed with a culture of *M. guilliermondii* 01 showed that oocyst integrity decreased as higher OD_600_ of culture were used. After 48 h of co-incubation, oocyst integrity had decreased to 53.90 % of its original value for OD_600_ of 1.0 (2.72 × 10^6^ CFU) of *M. guilliermondii*, whereas a decrease to 35.20 % was observed for OD_600_ 1.4 (6.72 × 10^6^ CFU). Controls (without yeast) at 24 and 48 h showed a decrease in integrity to 85.43 % and 82.09 % of the original values, respectively (Fig. 2). Statistical analysis showed significant difference among control and OD_600_ 1.0, 1.2, 1.4, 1.6 and 2.0 at 24 and 48 h (Fig. 2).

Controls (without yeast) at 24 and 48 h showed a decrease in integrity to 85.43 and 82.09 % of the original values, respectively (Fig. 2).

**Fig. 3:**
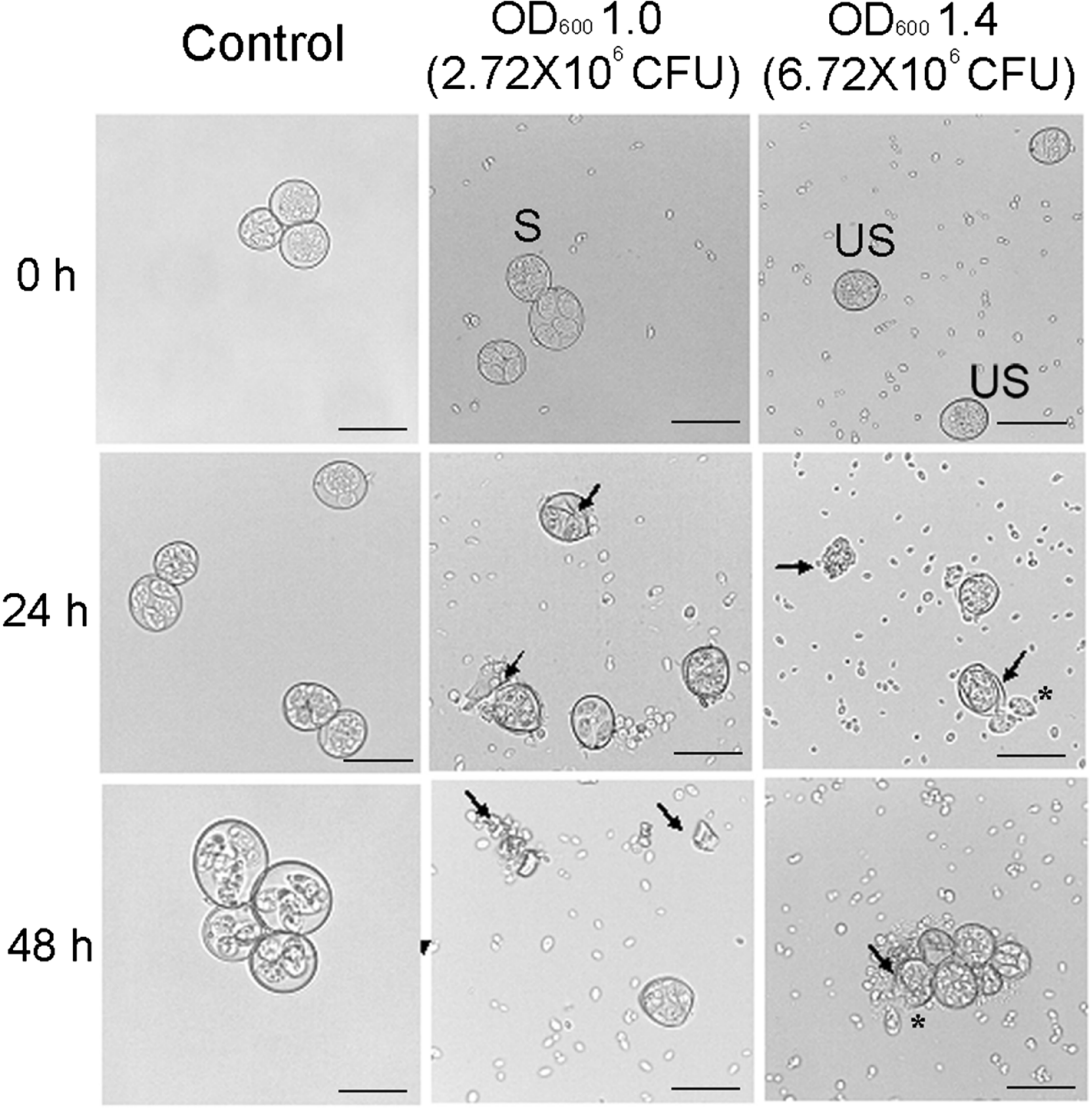
Microscopic observations of *Eimeria* spp. oocysts incubated with different numbers of *M. guilliermondii* 01 cells. After 24 h of incubation, damage to the oocyst wall is evident, and at 48 h, the damage is even greater, with cellular debris present. Control oocysts remain intact at the same incubation times. Cells of *M. guilliermondii* can be observed in the background. An OD_600_ of 1.0 corresponds 2.72 × 10^6^ CFUs, and an OD_600_ of 1.4 corresponds 6.72 × 10^6^ CFUs. Arrows indicate damaged oocysts. Asterisks indicate damaged sporocysts released from damaged oocysts. UN, unsporulated oocyst, S, sporulated oocyst. Scale bar, 150 μm

Damage to oocyst walls was observed microscopically. Control oocysts maintained their integrity; this is, displaying a double-layered structure with rounded, intact, light-reflective walls. Damaged oocysts displayed a structurally disturbed wall and in some cases showed sporozoites outside the oocyst. In most of the observations, a decrease was detected in the number of oocysts, and only structural lysis was observed (Fig. 3).

### Anticoccidial activity of culture, supernatant and ethyl acetate extract of *M. guilliermondii 01*

Oocyst wall integrity was assessed 0, 24 and 48 h after adding *M. guilliermondii* 01 supernatant, culture and ethyl acetate extract. It is noteworthy to mention that the ethyl acetate extract have been previously treated with trypsin and anticoccidial activity remained the same, which suggest that the compound with anticoccidial activity in the extract is not of protein nature.

**Fig. 4:**
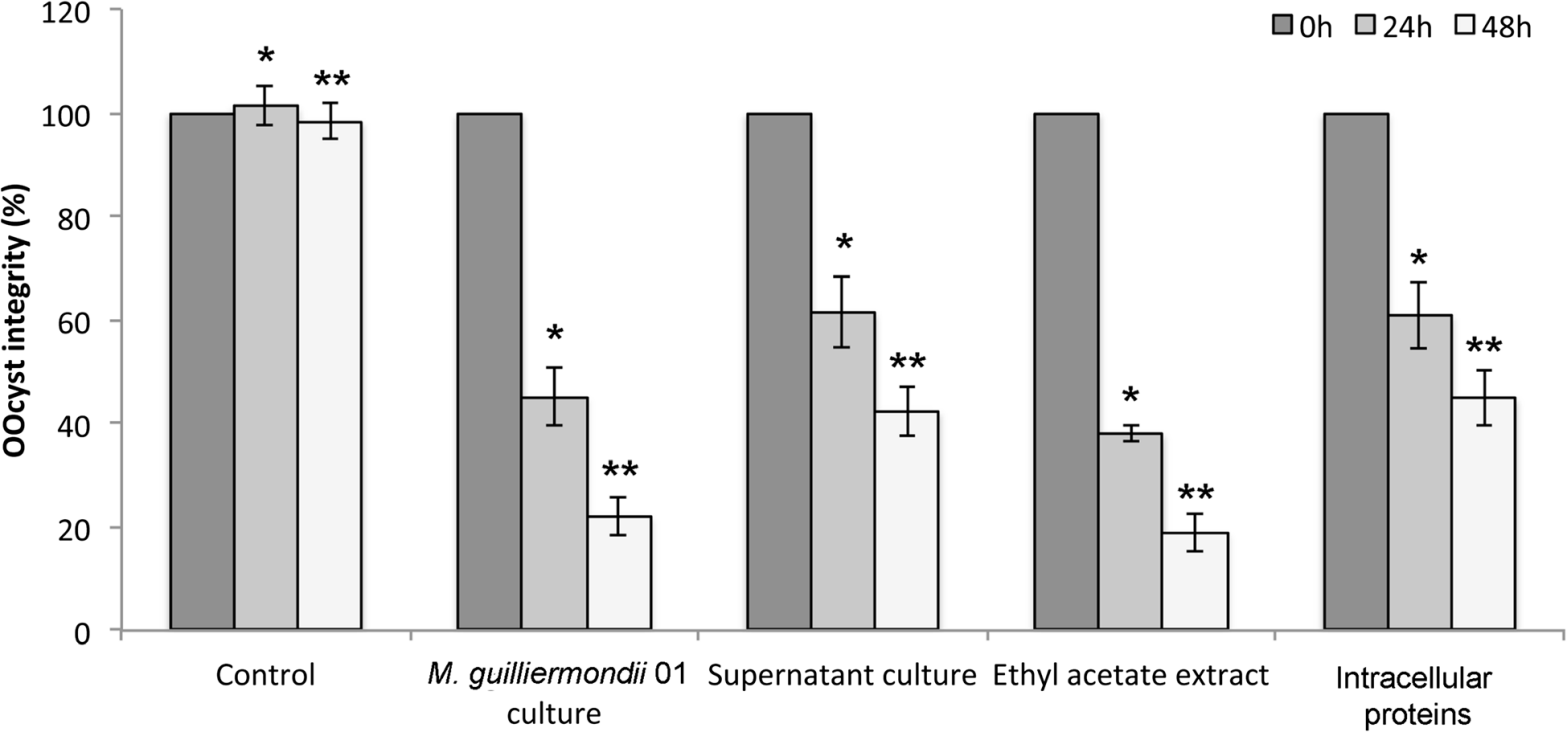
Comparison of the anticoccidial activities of *M. guilliermondii* 01 whole culture, culture supernatant, ethyl acetate extract and intracellular proteins on *E. tenella* oocyst After 48 h, the most severe damage of the oocyst wall was observed in bioassays with *M. guilliermondii* 01 culture and ethyl acetate extract showing 22.16 and 18.90 % of initial integrity, respectively. *M. guilliermondii* 01 intracellular proteins and supernatant culture also showed substantial anticoccidial activity, with 45 and 42.40 % of initial integrity after 48 h, respectively. This result suggests that anticoccidial activity is related to a excreted product and also an intracellular compound. Significant differences among treatments and control at 24 hare shown with an asterisk and significant differences among treatments and control at 48 h are shown with double asterisk (significance threshold, *P* < 0.05)

After 24 h of incubation, the anticoccidial activity of whole yeast culture on *Eimeria tenella* oocysts reduced their wall integrity to 45.16 %, whereas the integrity of oocyst walls after incubation with supernatant was 61.50 %. Oocyst integrity further decreased after 48 h of incubation, to 22.16 and 42.40 % of their original values with culture and supernatant, respectively. The integrity of the controls remained at 92.96 and 88.96 % after 24 and 48 h incubation, respectively (Fig. 4).

Ethyl acetate extracts of yeast culture supernatants showed anticoccidial activity against oocysts after 24 and 48 h of incubation. A 2.6-fold decrease in oocyst integrity was observed over the first 24 h of incubation (38.05 % integrity), and integrity further decreased by 2-fold between 24 and 48 h (18.90 % integrity) (Fig. 4).

### Anticoccidial activity of proteins

Anticoccidial activity of intracellular proteins was also assessed. After 24 h of incubation 61 % of oocysts maintained its integrity and decreased 1.3 fold after 48 h (Fig. 4).

**Fig 5:**
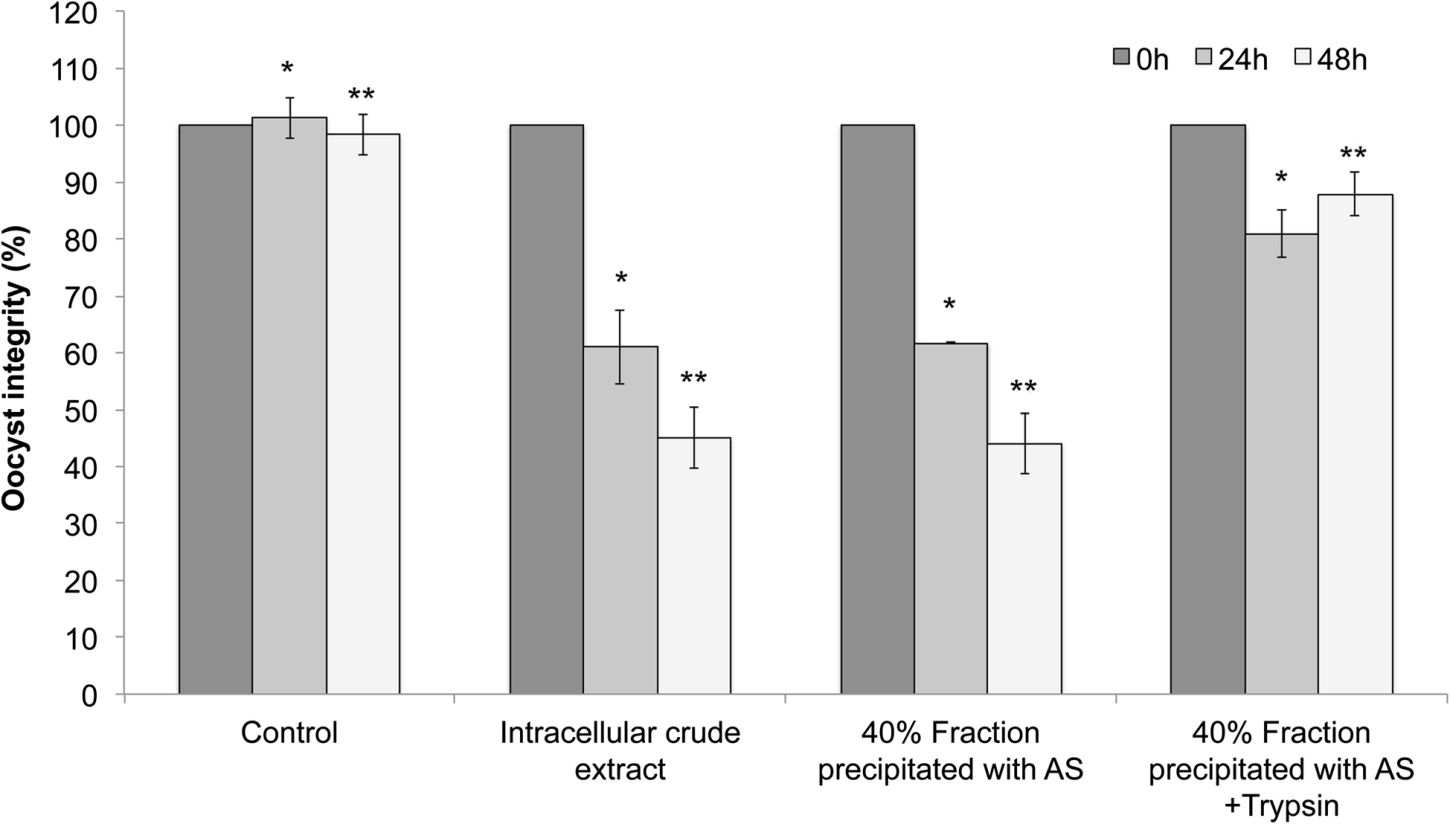
Anticoccidial activity of intracellular proteins. After 24 h of incubation with the intracellular crude extract, 61 % of *E. tenella* oocysts maintained their integrity in the intracellular crude extract, while in the protein precipitated with a 40 % fraction precipitated with ammonium sulfate (AS) 61.74 % of oocysts remained its integrity. After 24 h of incubation, 40 % precipitated with AS and treated with trypsin showed 80.92 % of integrity. The percentage of integrity decreased at 48 h to 45 and 44.06 % in intracellular crude extract and 40 % fraction precipitated with AS, respectively, whereas in the 40 % fraction treated with trypsin the integrity was 80.92 and 87.86 % at 24 and 48 h, respectively. . Significant differences among control and 24 h are shown with an asterisk while significant differences among control and 48 h and control are shown with double asterisk

The fraction precipitated with 40 % of ammonium sulfate maintained 61.74 % of oocyst integrity at 24 and 44.06 % of integrity at 48 h of incubation, whereas the same fraction treated with trypsin showed 80.92 and 87.86 % of integrity after 24 and 48 h of incubation, respectively (Fig. 5). No significant differences were observed between intracellular crude extract and 40 % fraction precipitated with ammonium sulfate, but a significant difference it was observed with respect to 40 % fraction precipitated with ammonium sulfate treated with trypsin. According to this, the effect of trypsin is clearly affecting the compound with anticoccidial activity present in the 40 % fraction.

## Discussion

The complex ITS regions ITS1 and ITS2 (non-coding and variable) and the 5.8S rRNA gene (coding and conserved) were useful for measuring close fungal phylogenetic relationships, as they exhibit far greater interspecific differences than the 18S and 26S rRNA genes [26].

Our in silico RFLP analysis of the 5.8S-ITS rDNA region showed a restriction pattern that matched the pattern reported for *Pichia guilliermondii* by [20]. In addition to this species, *Meyerozyma guilliermondii* was also found among the BLASTn results retrieved with the 5.8S-ITS rDNA region and D1/D2 domain.

The most common methods used for the identification of yeast species are based on the variability of the 5.8S, 18S and 26S ribosomal genes [27]. However, Kurtzman and Suzuki (2010) recently conducted a phylogenetic analysis of combinations of the D1/D2 domains of the large subunit and small subunit rRNA genes from budding ascomycetous yeast. These authors proposed five new genera: *Babjeviella, Millerozyma, Priceomyces, Scheffersomyces and Meyerozyma*. They proposed the name *Meyerozyma guilliermondii* (comb. nov. MycoBank no. MB513463) instead of the basionym *Pichia guilliermondii* [28].

Based on the phylogeny and other bioinformatic analyses, and because we have adopted this new proposed classification, we identified the anticoccidial yeast isolated from *Eimeria* oocysts as *Meyerozyma guilliermondii.*

The *in vitro* anticoccidial activity of this yeast was assessed in bioassays using *E. tenella* oocysts.

The effect of the active anticoccidial compound(s) was observed not only against *E. tenella* oocysts but also against other *Eimeria* species (*E. tenella, E. maxima, E. acervulina, E. praecox*) that showed similar results with respect to oocyst integrity and cell damage as is shown in Additional file 2.

We observed that increasing values of optical density (OD_600_) of yeast were associated with major damage to the oocyst, as higher OD_600_ counts in a bioassay corresponded to greater anticoccidial activity, a clearly dose-dependent effect. Besides, damage to the oocyst walls was evident 24 h after incubation and at 48 h, when cellular debris appeared. It is noteworthy that the compound(s) of *M. guilliermondii* culture can break the wall of sporulated and unsporulated oocysts.

This activity was observed not only with different OD_600_ values of whole yeast culture but also with culture supernatants. In this case, whole yeast culture and ethyl acetate extract showed the greatest activity against oocysts, with supernatant and intracellular crude extract showing less activity, indicating that the presence of yeast cells increases anticoccidial activity. Because activity was observed in whole yeast culture and in supernatants, the compound(s) that possess anticoccidial activity may be secreted by yeast.

When incubated *in vitro* with 2 mg/ml of the ethyl acetate extract for 24 h, we observed 61.95 % of damaged oocysts and 81.10 % at 48 h. Although the characteristics of the active anticoccidial compound(s) remain unknown, we suggest that it may have a polar nature because ethyl acetate has a polarity index of 4.4 and is generally considered a polar solvent (Fig. 4). Something similar was observed by A Remmal, S Achahbar, L Bouddine, N Chami and F Chami [16], they highlighted the activity of 4 mg/ml artemisia, thyme, clove and tea tree EO against 100,000 oocysts. After 25 h of incubation, this concentration induced approximately 80 % reduction in oocyst number.

Although essential oils showed a significant decrease in oocyst integrity, we only require half of the amount of our compound(s) extracted with ethyl acetate to observe a significant decrease in the number of oocysts. On the other hand, the economic factor involve in the production of essential oils make them an expensive alternative.

Recently, a product known as CitriStim (ADM Alliance Nutrition), that is a whole yeast co-product of citric acid extraction, containing the whole yeast and its components was used as a supplement in broiler production parameters and for immune responses during experimental coccidial infection. Researchers found that fecal oocysts diminished at 7 days post-coccidial infection, indicating accelerated clearance of coccidian [29].

We performed bioassays using CitriStim against *E. tenella* oocysts and against other species, but we did not find any significant damage to the oocyst walls or decreased numbers of intact oocysts after incubating for 24 and 48 h (data not shown).

The absence of activity by the yeast strain present in CitriStim could be explained by its identity as a different strain of *M. guilliermondii* that was possibly isolated from a different site.

A bioinformatic analysis performed (pairwise sequence alignment, EMBOSS Neddle) between 18S rDNA sequences showed only 73.5 % of identity between both yeasts. Studies of the genomic differences between these strains might elucidate the mechanism of anticoccidial action.

We analyzed the activity of our strain against different cells, including *Escherichia coli* K-12, *Saccharomyces cerevisiae* and KB human cells, but we did not find activity against any of them, suggesting that the activity is specific to *Eimeria*. This mayx not be the only activity of *M. guilliermondii* since it has been reported that the helvolic acid isolated from the culture of this yeast exerts an inhibitory activity on the spore germination of fungi *Magnaporthe oryzae* [30].

In our case, the obtained results are of great significance for future applications, especially after further in vivo analysis in chickens.

Although coccidiosis can be controlled with the use of anticoccidial drugs and with first-, second- and, recently, third-generation vaccines, none of these options are completely satisfactory; therefore, new alternatives are required. Our data show the effectiveness of *M. guilliermondii* 01 culture, culture supernatant and supernatant extract for damaging *E. tenella* oocysts. The results suggest that this yeast, isolated in oocyst suspensions from infected chickens, has important anticoccidial activity as shown in bioassays and could be used as a potent disinfectant of poultry environments.

An in vivo bioassay with chickens using anticoccidial compound(s) of *M. guilliermondii* 01 should be performed to enlighten the potential activity of this yeast. An interesting characteristic of *M. guilliermondii* 01 is that culture, supernatant or ethyl acetate extract are not the only that showed anticoccidial activity, but also intracellular proteins. In order to assess anticoccidial activity of excreted proteins we precipitated them from the supernatant and performed bioassays. However, no anticoccidial activity was observed (data not shown). The observed anticoccidial activity of intracellular crude extract suggests that the compound responsible of this activity is a protein. This was confirmed when we performed the bioassays using the precipitated proteins with 40 % of ammonium sulfate treated with trypsin. Additionally, we assessed the effect of trichloroacetic acid and heat on the 40 % fraction. After 24 and 48 h of incubation, 40 % fraction precipitated with TCA showed 93 and 99 % of integrity and the 40 % fraction that was heated showed 90 and 99 % of integrity at the same times as is shown in Additional file 3. This result supports the idea of a compound of proteic nature capable of destroy the oocyst wall, a two walls structure mainly composed by proteins, lipids and levels of carbohydrate covalently bounded to proteins, however, the composition of each layer is still unclear. If this compound cause damage to outer cell wall, it should be attacking a wall composed of quinone-tanned protein, as well as protein-tyrosine crosslinks [10].

We are now actively trying to identify the compound(s) that possess the observed anticoccidial activity in search of new products that can help to control the infection and dispersion stage of avian coccidiosis.

## Conclusions

We report here the damage to oocysts walls caused by *M. guilliermondii* culture, supernatant, supernatant extract and intracellular proteins. Our data from in vitro assays, suggest that anticoccidial activity of *M. guilliermondii* 01 is due to the excretion of a metabolite as well as proteic component. In both cases, a decreased number of oocysts were observed since a significant oocysts lost their viability because of the damage caused by the yeast products. The next step is to perform in vivo assays with the metabolite or protein treated oocyst, so far, the reported here shows the potential of this yeast and its products as a feasible method of coccidiosis control.

## Additional files

Additional file 1:
**Effect of DMSO on anticoccidial activity.** Bioassays were performed with *E. tenella* oocysts incubated with different percentages of DMSO (2 and 4 %) and ethyl acetate extract. Neither 2 nor 4 % of DMSO shown any effect on oocysts integrity after 48 h. On the contrary, oocysts treated with ethyl acetate extract showed almost 20 % of integrity after 48 h. This, strongly suggest that DMSO has a negligible effect on oocysts integrity. Significant differences among DMSO 2 and 4 % and ethyl acetate extract are shown with an asterisk; significant differences among DMSO 2 and 4 % and ethyl acetate extract are shown with double asterisk (significance threshold, *P* < 0.05). (TIFF 481 kb) Additional file 2:
**Anticoccidial activity of** ***M. guilliermondii*** **on** ***Eimeria*** **sp. oocyst.** After 48 h of incubation the anticoccidial activity values of the culture, supernatant, ethyl acetate extract and intracellular proteins decreased 1.17, 1.28, 1.26 and 1.02-fold, respectively. There are not significant differences between treatments (significance threshold, *P* < 0.05). (TIFF 94 kb) Additional file 3:
**Anticoccidial activity of 40 % fraction treated with trypsin or heat.** 40 % fraction precipitated with ammonium sulfate and treated with TCA showed similar values of integrity that heated 40 % fraction. There are not significant differences between treatments (significance threshold, *P* < 0.05). (TIFF 237 kb)

## Abbreviations

mm: Milimeters
g: Grams
L: Liter
h: Hours
ITS: Intergenic Sequence
rDNA: Ribosomal DNA
rRNDAm: Ribosomal RNA
OD: Optical density
CFU: Colony-Forming Unit
ml: Mililiters
μl: Microliters
kD: KiloDalton
mM: Milimolar
μg: Micrograms
rpm: Revolutions per minute
