# Supplementary figures and images for: Impact of Meyerozyma guilliermondii isolated from chickens against Eimeria sp. protozoan, an in vitro analysis
Source: BMC Vet Res. 2015 Nov 9;11:278. doi: 10.1186/s12917-015-0589-0 (PMC4640389; doi:10.1186/s12917-015-0589-0)

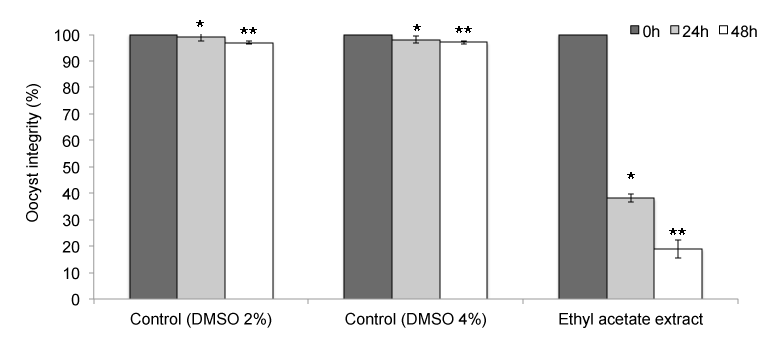

Supplement: Additional file 1: — Effect of DMSO on anticoccidial activity. Bioassays were performed with E. tenella oocysts incubated with different percentages of DMSO (2 and 4 %) and ethyl acetate extract. Neither 2 nor 4 % of DMSO shown any effect on oocysts integrity after 48 h. On the contrary, oocysts treated with ethyl acetate extract showed almost 20 % of integrity after 48 h. This, strongly suggest that DMSO has a negligible effect on oocysts integrity. Significant differences among DMSO 2 and 4 % and ethyl acetate extract are shown with an asterisk; significant differences among DMSO 2 and 4 % and ethyl acetate extract are shown with double asterisk (significance threshold, P < 0.05). (TIFF 481 kb) [file 12917_2015_589_MOESM1_ESM.tiff]

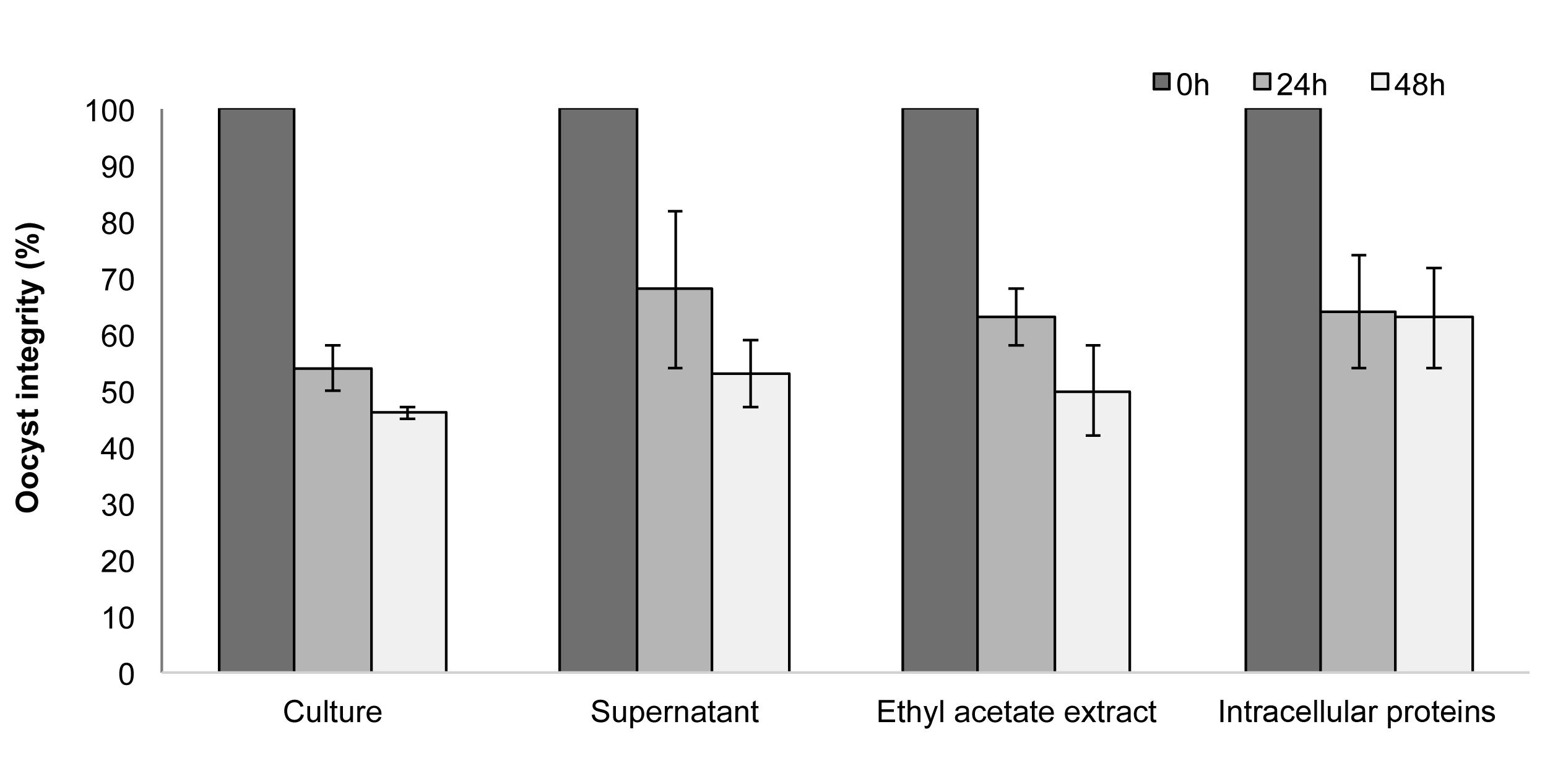

Supplement: Additional file 2: — Anticoccidial activity of M. guilliermondii on Eimeria sp. oocyst. After 48 h of incubation the anticoccidial activity values of the culture, supernatant, ethyl acetate extract and intracellular proteins decreased 1.17, 1.28, 1.26 and 1.02-fold, respectively. There are not significant differences between treatments (significance threshold, P < 0.05). (TIFF 94 kb) [file 12917_2015_589_MOESM2_ESM.tiff]

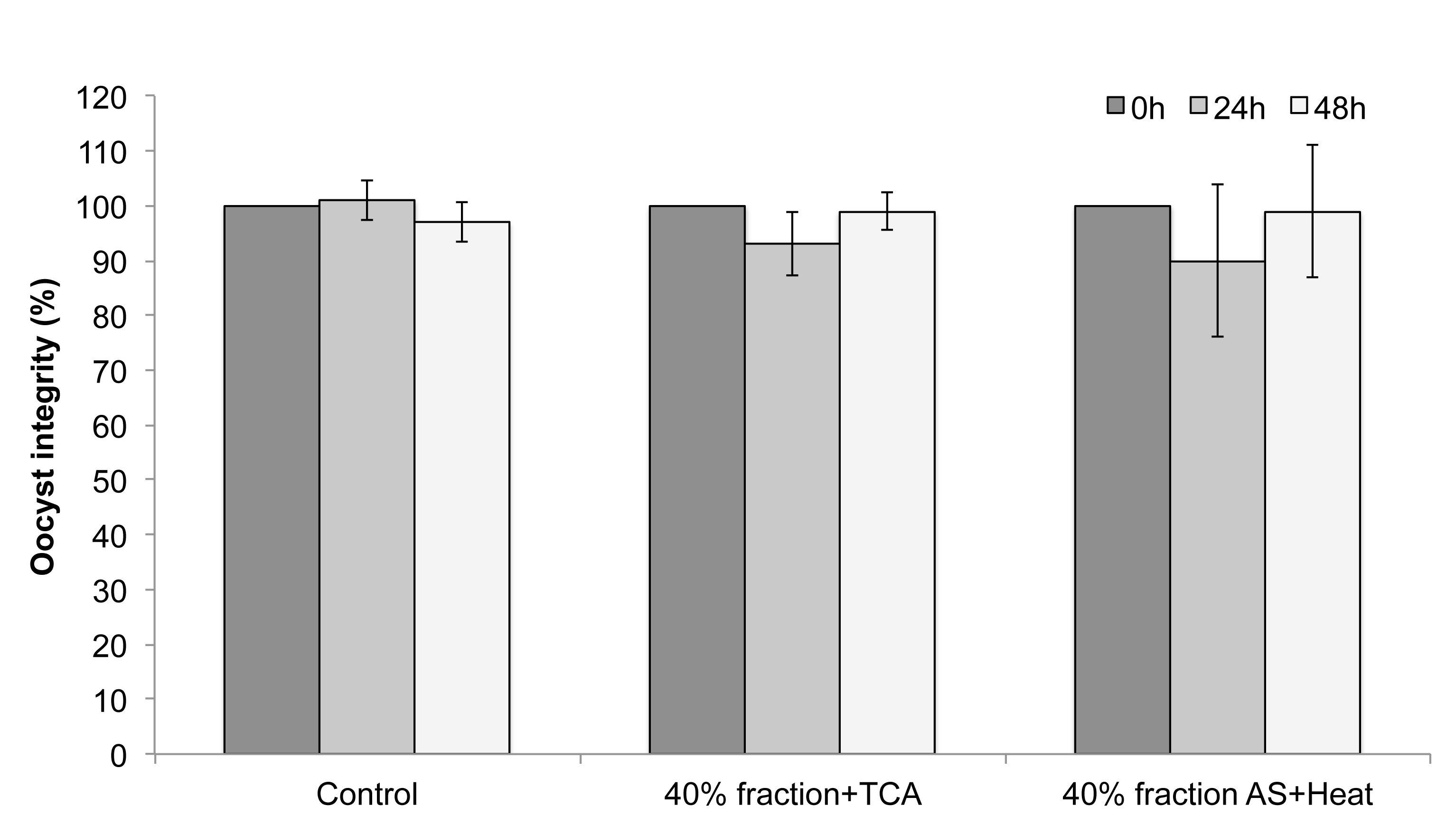

Supplement: Additional file 3: — Anticoccidial activity of 40 % fraction treated with trypsin or heat. 40 % fraction precipitated with ammonium sulfate and treated with TCA showed similar values of integrity that heated 40 % fraction. There are not significant differences between treatments (significance threshold, P < 0.05). (TIFF 237 kb) [file 12917_2015_589_MOESM3_ESM.tiff]
